# Chemically Modified Plastic Tube for High Volume Removal and Collection of Circulating Tumor Cells

**DOI:** 10.1371/journal.pone.0133194

**Published:** 2015-07-15

**Authors:** Angelo Gaitas, Gwangseong Kim

**Affiliations:** 1 Kytaro, Inc., Miami, Florida, 33199, United States of America; 2 Florida International University (FIU), Miami, Florida, 33199, United States of America; Sapporo Medical University, JAPAN

## Abstract

In this preliminary effort, we use a commercially available and chemically modified tube to selectively capture circulating tumor cells (CTCs) from the blood stream by immobilizing human anti-EpCAM antibodies on the tube's interior surface. We describe the requisite and critical steps required to modify a tube into a cancer cell-capturing device. Using these simple modifications, we were able to capture or entrap about 85% of cancer cells from suspension and 44% of cancer cells from spiked whole blood. We also found that the percentage of cells captured was dependent on the tube's length and also the number of cancer cells present. It is our strong belief that with the utilization of appropriate tube lengths and procedures, we can ensure capture and removal of nearly the entire CTC population in whole blood. Importantly after a patient’s entire blood volume has circulated through the tube, the tube can then be trypsinized to release the captured live CTCs for further analysis and testing.

## Introduction

During cancer metastasis, the cells detach from the primary tumor detach, circulate in the bloodstream via the circulatory system and get lodged at tissues distant from the primary site where they begin to grow and multiply giving rise to secondary tumors. Current treatments have been largely ineffective in treating metastasis, as is evident by the fact that more than 90% of cancer deaths are due to metastasis [1]. Conventional treatments such as chemotherapy and radiation therapy are severe and have, in many cases, toxic side effects. Further, recent evidence supports the "tumor self-seeding" concept, in which circulating tumor cells (CTCs) colonize an existing tumor, thus increasing its aggressiveness [2].

CTCs are generally believed to play a significant role in the metastatic process. In the past decade, research has concentrated on developing methodologies for the detection, enrichment, and enumeration of CTCs for diagnostic purposes. These efforts include micro-fluidic separation devices [3–5], devices that rely on size exclusion by centrifugation [6, 7] or filtration [8, 9], immuno-magnetic separation [10, 11] and fluorescence-activated cell sorting (FACS) technologies [3, 12] and several other techniques or combinations thereof. These techniques are generally referred as "liquid biopsy" [4, 13]. In liquid biopsy, a small blood sample is drawn from a patient and analyzed ex-vivo for CTCs. CTCs are typically separated and purified by antibodies, such as the epithelial cell adhesion molecule (EpCAM) [14], cytokeratins [15], to name a few, and subsequently enumerated. These numbers are indicative of the progression of the disease. However all the aforementioned techniques are constrained by the low volume extracted for analysis and thus by the low number of CTCs that can be detected. Other efforts have targeted larger blood volumes; for example, one study incorporated the use of a structured and functionalized medical wire coated with anti-EpCAM to enrich CTC from larger volumes [16].

A lot of experimental data suggests that techniques that remove CTCs from blood circulation *in vivo* could reduce metastatic events, and at the same time reducing the aggressiveness of existing tumors. There is indirect evidence that blood filtering, such as hemodialysis, might reduce cancer metastasis by removing circulating tumor cells (CTCs) from the bloodstream [17–19]. Extracorporeal filtration devices using leukocyte depletion filters have been used during surgical removal of tumor cells in order to reduce the risk of their dissemination [20–22]; however, these devices have not been used to reduce metastasis post-surgery and therefore putting a patient at risk of recurrent replapses. There have been efforts to remove or kill cancer cells using microtubes functionalized with antibodies, selectin and TRAIL with a capture and a kill rate between 30–41% [23, 24]. Recently, a technique to kill cancer cells in the bloodstream was demonstrated by functionalizing circulating leukocytes with cancer-specific TNF-related apoptosis inducing ligand (TRAIL) and E-selectin adhesion receptor [25].

**Fig 1:**
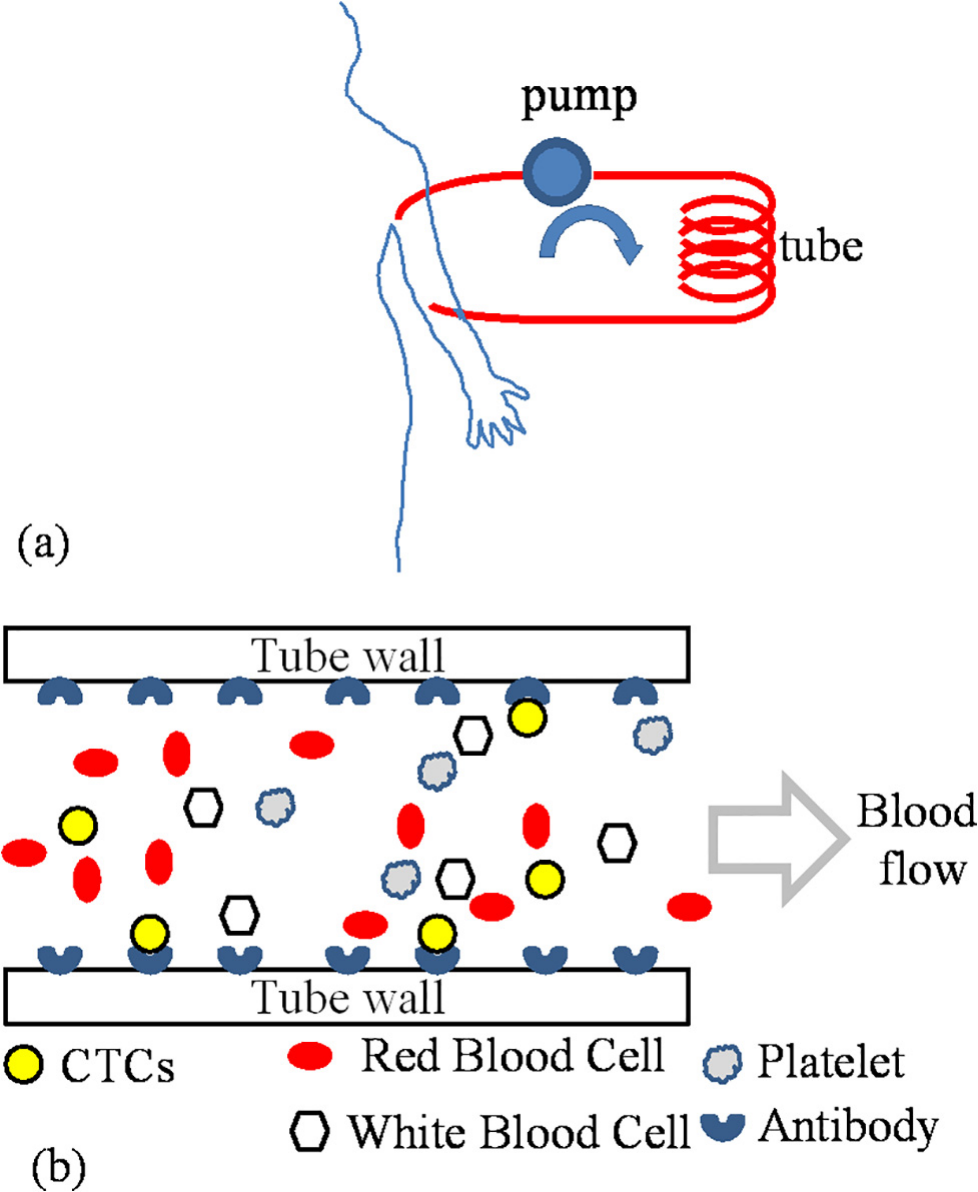
(a) Conceptual diagram of extracorporeal device. (b) CTC capturing by antibody immobilized on tube.

In this preliminary work, we put forward a simple method that employs an extracorporeal tube to remove and collect CTCs from the bloodstream with potential applications in: (a) Reducing metastasis by removing CTCs from circulation and (b) In diagnostic applications such as CTC enumeration and genetic analysis. Our device consists of a modified commercially available plastic tube that is functionalized with EpCAM antibodies. EpCAM is a widely used CTC marker [14]. At this proof-of-concept stage, the device already exhibits improved capturing efficiency coupled with the fact that the device has a simple design, it is, inexpensive, and finally able to handle large volumes of whole blood without the need of separation and processing procedures (Fig 1(A)). Our method does not introduce any foreign agents into the bloodstream and entails aseptic procedures; instead, blood flows through a tube in which CTCs bind to appropriate antibodies (such as EpCAM) coated on the inner surface of the tube.

## Materials and Methods

### Tube surface modification

**Fig 2:**
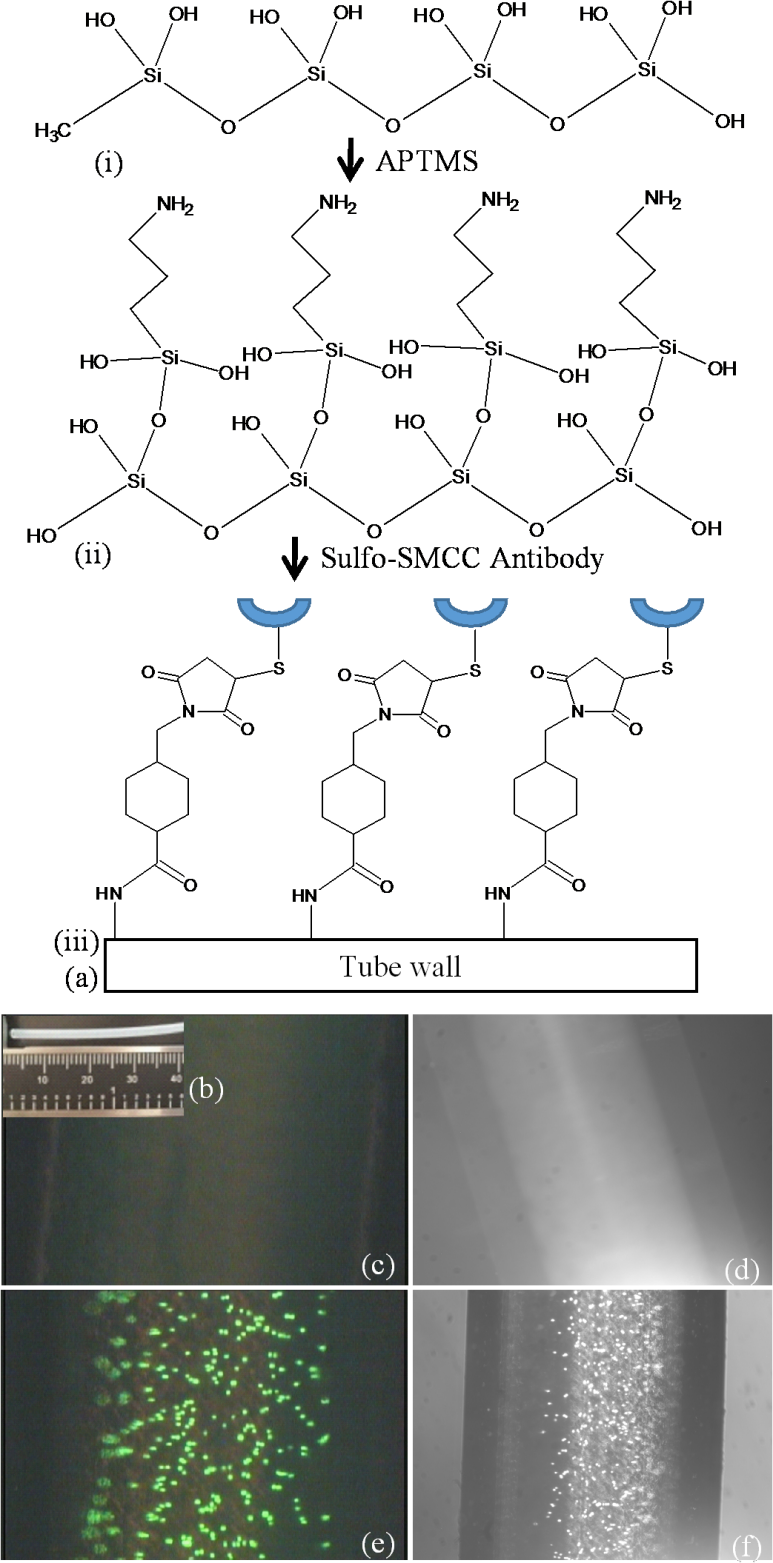
(a) Schematic of the tube preparation: (i) activation of PDMS tube surface; (ii) amine functionalized tube surface; (iii) antibody conjugation. (b) Photomicrograph of the tube, were functionalized with human anti-EpCAM (Scale bar in mm). (c & d) PC-3 cells were placed in an unmodified tube (without EpCAM coating), as control experiments, demonstrating absence of any captured cells in the cavity of the tube. (d) (e) Fluorescence microscopic images of captured PC-3 cells on anti-EpCAM immobilized tube and (f) phase contrast image of the same PC-3 cells.

A polydimethylsiloxane (PDMS) tubing (Dow Corning Silastic laboratory tubing with an internal diameter of 1.02 mm) was chosen for this study (Fig 2(B). The tube length was about 120 cm for experiments entailing cancer cell suspensions and 15 cm for whole blood tests. The tube’s internal surface was activated by treatment with an acidic hydrogen peroxide solution (H_2_O:HCl:H_2_O_2_ in 5:1:1 volume ratio) for five minutes at room temperature [26]. The tube was rinsed with excess deionized (DI) water five times and dried in air. This treatment formed the hydrophilic surface with hydroxyl groups (-OH) available for further functionalization (Fig 2(A) (i)). The tube was then filled with aminopropyltrimethoxysilane (APTMS) for 10 minutes (Fig 2(A) (ii))). The tube was rinsed with excess amount of DI water at least five times and dried in air. This step adds the primary amine group on the surface based on the sol-gel reaction principle [27, 28]. To verify the presence of the primary amine group on the tube surface, a short section of the treated tube was filled with an amine reactive fluorescence dye, fluorescein isothiocyanate (FITC, 0.1 mg/mL in PBS pH 7.4) for one hour (Fig 2(A) (ii)). The tube was then rinsed and the fluorescence from its inner surface was monitored using a fluorescence microscope.

### Immobilization of anti-EpCAM on the surface of the tube

Phycoerythrin (PE)—labeled human EpCAM (eBiosciences) antibody was treated for one hour with Traut’s reagent (2-iminothiolane HCl, 2-IT) to generate an available sulfhydryl group (-SH) (anti-EpCAM:2-IT = 1:10 in mole ratio) in PBS (pH 7.4). Then, unbound 2-IT was removed from the antibodies using a spin column (MW 30 kDa, cutoff, Amicon filter or Corning Spin-X protein concentrator) at 4000 RCF for 30 minutes. The concentrated anti-EpCAM was re-suspended in PBS, and the volume adjusted to of 1 mL. During the antibody-2-IT reaction, the amine functionalized tube was filled with a hetero-bifunctional (amine reactive at one terminal and thiol reactive at the other terminal) cross-linker, sulfo-SMCC (sulfosuccinimidyl 4-[N-maleimidomethyl]cyclohexane-1-carboxylate) in 2 mg/mL concentration in PBS (pH 7.4). After the EpCAM was spun down, the sulfo-SMCC solution was removed and the tube was rinsed in PBS and re-filled with 1 mL EpCAM solution. The reaction was run on a shaker for two hours at room temperature and continued overnight at 4°C. The next day, after the unbound EpCAM solution was collected, the tube was gently rinsed with PBS and then refilled with 1 mg/mL L-cystein for another two hours (Fig 2(A) (iii)). The conjugation of anti-EpCAM on the tube surface was confirmed by PE's fluorescence on a fluorescence microscope.

### Cell culture

A human prostate cancer cell line, PC-3, was purchased from American Type Culture Collection (ATCC) and propagated in RPMI 1640 media supplemented with 10% fetal bovine serum and 1% Penicillin-Streptomycin. The expression of EpCAM on the cell surface was positively confirmed by performing the reaction with PE-labeled EpCAM and monitoring the fluorescence label on the cell surface.

### Cell capturing in cell suspension

PC-3 cells were detached from the culture flask by treatment with 0.25% trypsin-EDTA. The cell density was determined using a hemocytometer. 250,000 cells in 1 mL RPMI were selected for the capturing experiments. The anti-EpCAM immobilized tube was filled with 1 mL PC-3 cell suspension. The tube was incubated at room temperature for one hour; the cell solution was then collected from the tube and the number of cells remaining in the collected solution were again measured by a hemocytometer. The capturing efficiency was calculated using the following equation (Initial cell density–final cell density)/initial cell density x 100 (%). After removing the cell solution, the tube was refilled with cell media containing a live cell fluorescence marker, Calcein AM. The captured cells inside tube were imaged by fluorescence microscopy based on Calcein AM’s fluorescence at 4 x objective magnification. A control experiment was performed in parallel using another silicone tube without surface modification and antibody immobilization. All the sample solutions were processed for flow cytometry.

### Processing samples for flow cytometry

All the samples collected through capturing experiments were pelleted by centrifugation at 1000 RPM for 5 min. The supernatant was removed and the cell pellet was re-suspended in 4% glutaraldehyde in PBS for fixation (PC-3 cells only in equal volume (1 mL) and PC-3 cells in blood in 10x excess volume (10 mL)). Samples were then gently agitated for 15 minutes at room temperature. The samples were centrifuged again to remove excess fixative. The supernatant was removed and fixed cells were re-suspended in PBS. Once again, a centrifugation and re-suspension cycle was run for washing. PC-3 only samples were adjusted to 1 mL final volume while PC-3 in blood samples were diluted in 10 mL (10x dilution) with PBS; fixed specimens were kept refrigerated until flow cytometry.

### Flow cytometry

Further evaluation of cell capture was investigated using flow cytometry to determine the cell capture before and after exposure to the anti-EpCAM immobilized tube. PC-3 in media samples were measured using a Milteny MACSQuant device based on scattering (SSC vs FSC) because the cells were not labeled by any fluorescence tag and this device could provide data based on actual cell count / volume. 50 μL were injected for the measurements.

### Cell capturing in blood

Human whole blood was purchased from Innovative Research (www.innov-research.com). PC-3 cells were trypsinized for suspension and the initial cell density was measured using a hemocytometer. After cell counting, cells were stained with Calcein AM for 15 min at 37°C, after which PC-3 cells were centrifuged at 1000 RPM for five minutes to exclude free Cacein AM from suspension. After removing the supernatant, the cell pellet was re-suspended in RPMI media to achieve a density of 50,000 cells/20 μL. 20 μL of Calcein AM labeled PC-3 cells were added to 100 μL whole blood aliquots containing various anti-coagulants (Li-heparin, K2-EDTA, Na-citrate), and in blood with no anti-coagulants. Prepared EpCAM immobilized tubes (15 cm long) were filled with PC-3 spiked whole blood and incubated for two hours on a shaker at room temperature ((Fig 1(B)). The solutions were collected and processed for cell counting. Six replicated sets of PC-3 spiked whole blood samples were prepared, including three for cell count determination before tube (initial count) and three for cell count determination after capturing in tube (final count). The cell counts before and after were determined by a hemocytometer based on green fluorescence from Calcein AM staining to discriminate PC-3 cells from other blood cells.

## Results and Discussion

The images in Fig 2E & 2F are of PC-3 cells captured by anti-EpCAM conjugated silicone (PDMS) tube after 1 hour of incubation. After collecting the solution from the tube, the captured cells were stained with Calcein AM containing cell media and imaged using a GFP filter cube (Ex: 485 nm / Em: 525 nm) in an Olympus IMT-2 fluorescence microscope. The result showed that PC-3 cells were effectively captured by the anti-EpCAM immobilized tube. As Calcein AM is also a cell viability fluorescence probe, these images also confirmed that the captured cells are alive. In contrast, the unmodified control tubes, shown in Fig 2C & 2D, exhibited negligible capture of PC-3 cells.

**Fig 3:**
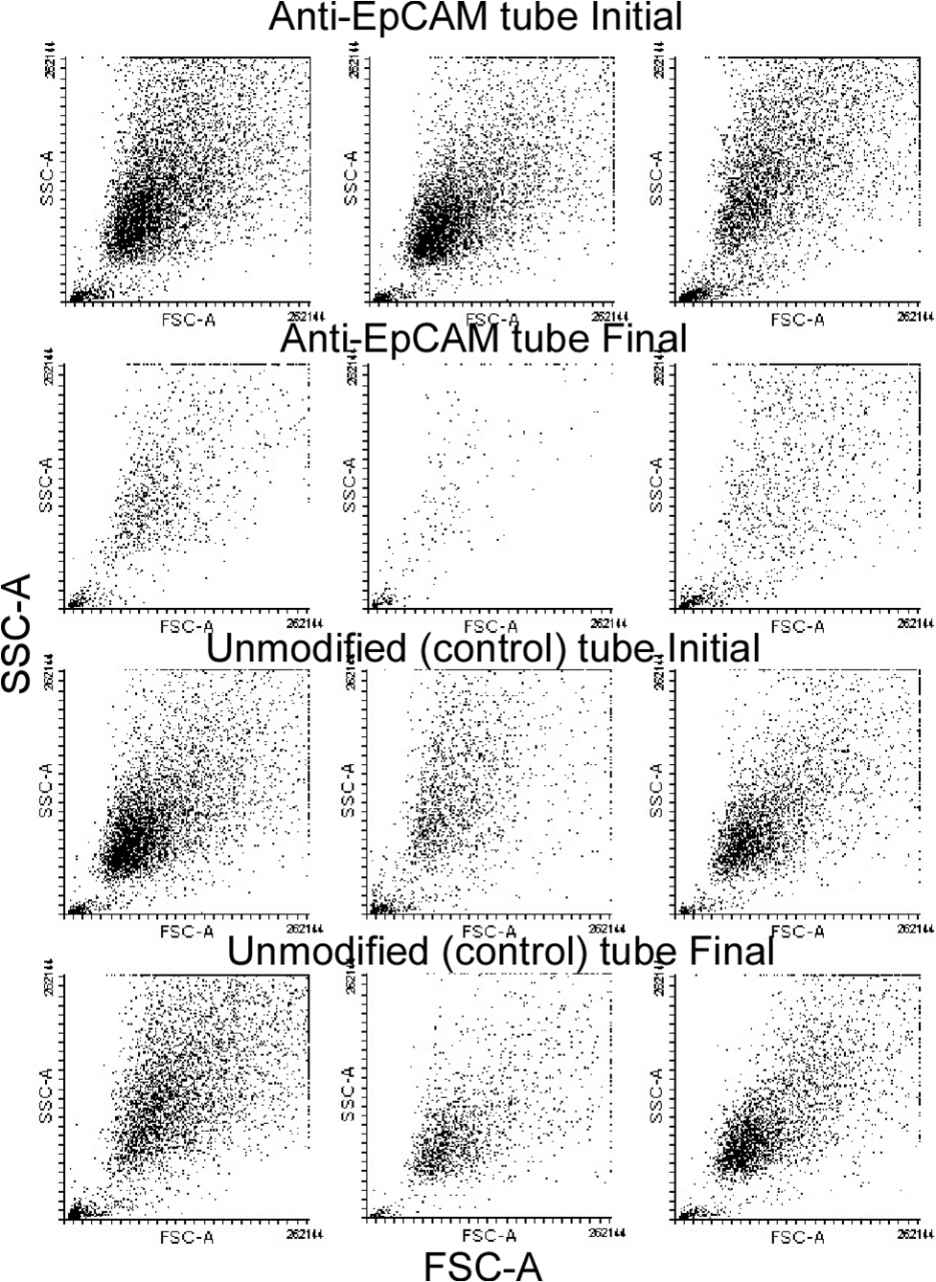
Representative flow cytometry dot plots. The figures in the two upper panel rows clearly demonstrated the reduction in cell count by anti-EpCAM immobilized tubes, while the figures in the two lower panel rows showed minor difference between initial and final count by unmodified tube used as a control.

The cell capture was quantitatively determined by using a hemocytometrer (Table 1) and flow cytometry (Table 2 and Fig 3). These results clearly demonstrated that target cells (PC-3) can be captured in a highly effective manner, resulting in a significantly reduced number of cancer cells in the media (about 85%, confirmed by both counts in a hemocytometer and flow cytometry). The unmodified tube captured a significantly lower number of cells (about 0.8% in hemocytometry and 5.7% in flow cytometry). This indicates that slight non-specific binding occurred during the 1 hour of exposure to the unmodified tube surface. Non-specific binding can be further reduced by surface modification using, for example, a PEGylation (polyethyleneglycol) coating.

**Table 1:** Cell count estimation by hemocytometry. The values are of mean ± standard error of the mean (n = 3). P<0.001 of control vs. immobilized tube *t-test*.

Samples	Initial (# cell/mL)	Final (# cell/mL)	Capture %
PC-3 in EpCAM immobilized tube	253,333 ± 2,083	38,750 ± 9,922	~85
PC-3 in unmodified tube (Control)	257,917 ± 2,887	255,833 ± 5,774	~1

**Table 2:** Cell count estimation by flow cytometry. The values are of mean ± standard error of the mean (n = 3). *P<* 0.01 of control vs. immobilized tube *t-test*.

Samples	Initial (# cell/mL)	Final (# cell/mL)	Capture %
PC-3 in EpCAM immobilized tube	93,467 ± 13,360	12,907 ± 5,153	~85
PC-3 in unmodified tube (Control)	61,440 ± 18,920	56,646 ± 15,000	~5.7

**Fig 4:**
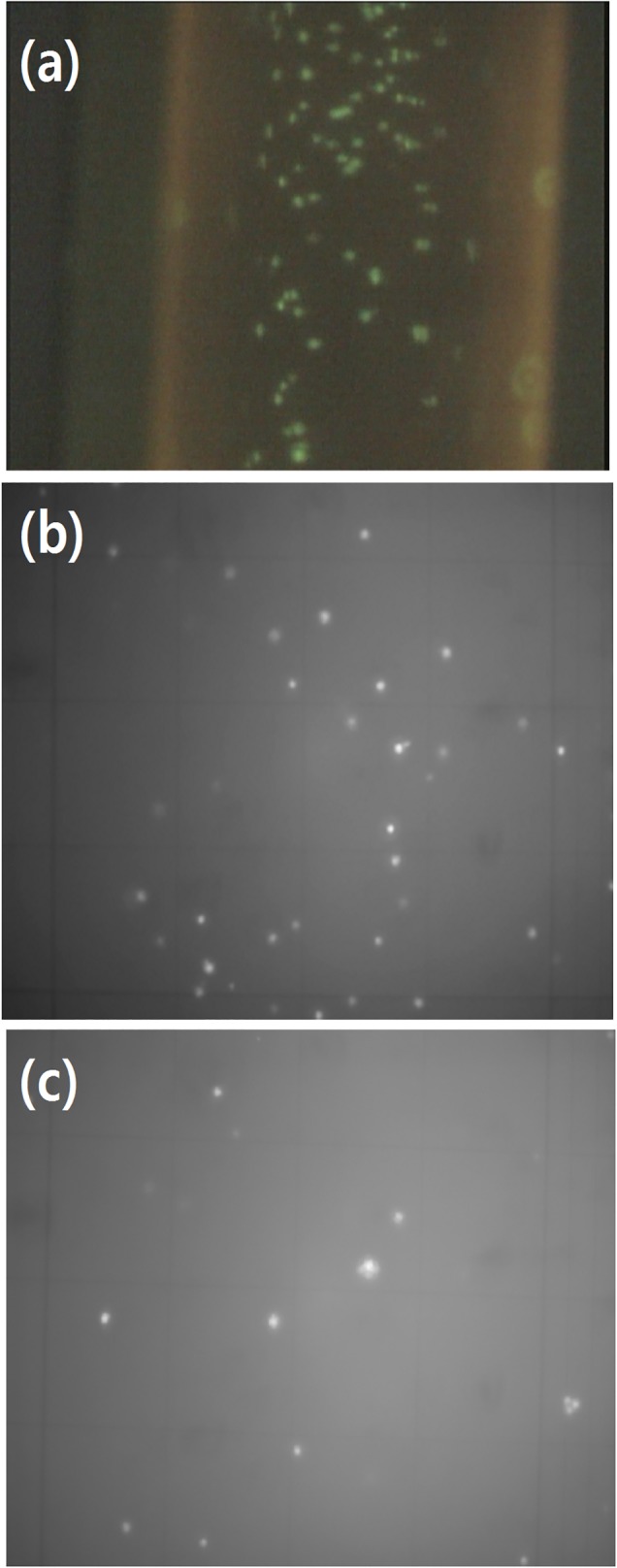
(a) Fluorescence microscopy images for captured PC-3 cells on anti-EpCAM immoibized tubes in the presence of whole blood. The PC-3 cells were pre-stained by Calcein AM and Na-citrate was used as an anticoagulant for the blood sample. (b) and (c) are representative images PC-3 cells from the hemocytometry counting using Calcein AM fluorescence. Initial (b) showed significantly more cells than final (c), indicating reduction of cell density by capture.

The EpCAM immobilized tubes showed about 44% capture efficiency of PC-3 cells in whole blood (Table 3 and Fig 4). The reduction in capture efficiency confirms the existing complications in CTC capturing in blood. Blood is a suspension that is enriched with several cell populations, including red blood cells, white blood cells, platelets, and other vesicles. The presence of these blood components could limit the movement of PC-3 cells in the solution and reduce the chances of PC-3 cells having contact with anti-EpCAM on the tube's inner surface.

**Table 3:** Quantitative analysis of cell capture from hemocytometry. Three independent experiments exhibited an average of 44.7% capture efficiency in PC-3 cell spiked whole blood system. *P<* 0.01 *initial vs*. *final*, *t-test*.

Count/ 100 μL	Initial 1	Initial 2	average Initial	final 1	final 2	average final	capture efficiency
trial 1	29000	31500	30250	14750	15750	15250	49.6%
trial 2	27500	22750	25125	16500	17250	16875	32.8%
trial 3	27000	21750	24375	10250	13250	11750	51.8%
Average	26583 ± 1846			14625 ± 1512			44.7%

Secondly, we found that anti-coagulants can significantly interfere with the binding of EpCAM antibody to the targeted PC-3 cells. In order to avoid blood clotting during the capture experiments, which could inhibit the movement of cells by gelation and also clog the tube, anti-coagulants are necessary. It is also known that platelets aggravate CTC metastasis; therefore, the use of anticoagulants may aid in the reduction of metastatic events [29]. We originally used Li-heparin as an anti-coagulant and consistently obtained negligible capture of PC-3 cells. We confirmed the lack of captured cells by fluorescence imaging and by hemocytometry. While exploring solutions for this problem, we found that the capturing experiment using whole blood without any anti-coagulant showed positive cell capture despite interference by clotting. The presence of anti-coagulants was suspected of being responsible for the inhibition of affinity to anti-EpCAM. Two additional anti-coagulants, Na-citrate and K_2_-EDTA, were studied (shown in S1 Fig). The results revealed that K_2_-EDTA also inhibited cell capturing, while Na-citrate allowed cell binding. It is still not certain whether Na-citrate does not interfere with binding or does so partially. We were unable to find any interfering effects anti-coagulants may have in CTC detection/diagnosis studies in published literature.

Third, we discovered that other antibodies can impede the anti-EpCAM to PC-3 binding. In order to estimate the cell density change in the presence of blood, we initially used dye conjugated CD-44 antibody to visualize the PC-3 cells. CD-44 expression in PC-3 cells has been previously reported [30]. The capturing efficiency of anti-EpCAM immobilized tubes between CD-44 labeled PC-3 cells and PC-3 with no label was compared; this revealed that the number of captured cells was reduced by half when the PC-3 cells were labeled by CD-44 as compared to PC-3 cells with no label (S2 Fig and Table 1). These results indicated that CD-44 interferes with the EpCAM antibody-antigen binding. EpCAM is known to often associate with other proteins, including claudin-7, tetraspanin or CD44, etc., and a possible explanation could be that EpCAM affinity can be inhibited by co-existence of a high affinity antibody [31]. It is not known whether CD-44 antibody and EpCAM have a common epitope to compete with each other in PC-3 cells. This result also allows the inference that other isolation technologies that rely on multiple antibodies may be influenced by this effect.

We believe that capture efficiency can be further improved. While we discovered the aforementioned complications in whole blood, we reduced the tube length for the experiment from 120 cm tube for PC-3 suspension study to 15 cm tube for capturing in blood. However, the number of cells could not be reduced sufficiently to maintain reliable numbers in hemocytometer counting (250,000 cells for suspension study to 50,000 cells for blood study). Considering the ratio of the number of captured cell over the tube length, the capturing efficiency in the blood study was only 17% less than that in suspension (1,770 cells/cm vs 1,467 cells/cm). The capturing efficiency can be improved by using a longer tube, thus making it necessary to optimize the tube length, dependent on the blood volume required to cleanse. Unlike other CTC detection/diagnostics technologies that concentrate on small volumes and enrich CTCs, the tube format is not necessarily limited by sample volume. Eventually, an unlimited volume of samples can be circulated through the tube, thus removing the majority of CTCs from blood. We believe that this procedure can be done safely and successfully in a clinical setting by processing the entire blood in continuous or intermittent flow. Alternatively, consecutive drawings of as much as 0.5 liter of blood (a quantity in line with typical blood donations) can undergo the cleaning process for CTC removal. The blood can then be re-injected in the patient as the whole procedure can be performed under aseptic conditions. The process can be repeated until all of the blood is cleared of CTCs (a typical adult has a blood volume of between 4.7 and 5 liters).

We also confirmed that, by removing captured cells by trypsinization (data not included), the tubes can be reused with only a minor reduction in capturing ability This method can be easily adapted to current medical practices using existing medical tubing without the need for complicated microfluidics and micro-fabrication.

While, in these experiments, we used EpCAM, we do recognize that other types of cancer cells that have undergone epithelial-to-mesenchymal transition (EMT) and do not express epithelial markers on their surfaces. Thus, additional markers would have to be added in the future.

The device can be used for diagnostics as well as therapeutic applications, given that the captured cells on the tube can be counted and further re-suspended and geneotyped. An interesting application of this technique is in collecting live cancer cells for patient-derived tumor xenografts (PDTX) models to be used in cancer drug research. Additional filters and apoptosis- causing agents may also be added to enhance the capture/kill rate. This device can be used during tumor surgery to remove CTCs to reduce the risk of their spreading through the blood. Finally, we want to note that this principle can also be applied to other conditions such as bacterial or viral blood borne infections.

## Supporting Information

S1 Fig The effect of anticoagulants on cell capture. Cell capture was significantly reduced in the blood when Li-heparin and K2-EDTA were used. Successful cell capture was observed in blood with Na-citrate. The cell capturing in the blood without any anti-coagulant was examined by removing early blood clots from blood specimen. The anti-EpCAM immobilized tube could effectively capture the cells in this blood sample.
(TIF)

S2 Fig The effect of additional antibodies on cell capturing. PC-3 cells were labeled by FITC conjugated CD-44 antibodies prior to the cell capturing experiment in the blood. Captured cells were re-stained after capture by filling the tube with Calcein AM contained RPMI media. Compared to PC-3 cell without prior labeling (left), cell capture in CD-44 pre-stained PC-3 was considerably reduced (right).
(TIF)

S1 Table The reduction in capture efficiency by CD-44 pre-staining was quantified using hemocytometry. (PDF)
