# Supplementary figures and images for: Chemically Modified Plastic Tube for High Volume Removal and Collection of Circulating Tumor Cells
Source: PLoS One. 2015 Jul 15;10(7):e0133194. doi: 10.1371/journal.pone.0133194 (PMC4503618; doi:10.1371/journal.pone.0133194)

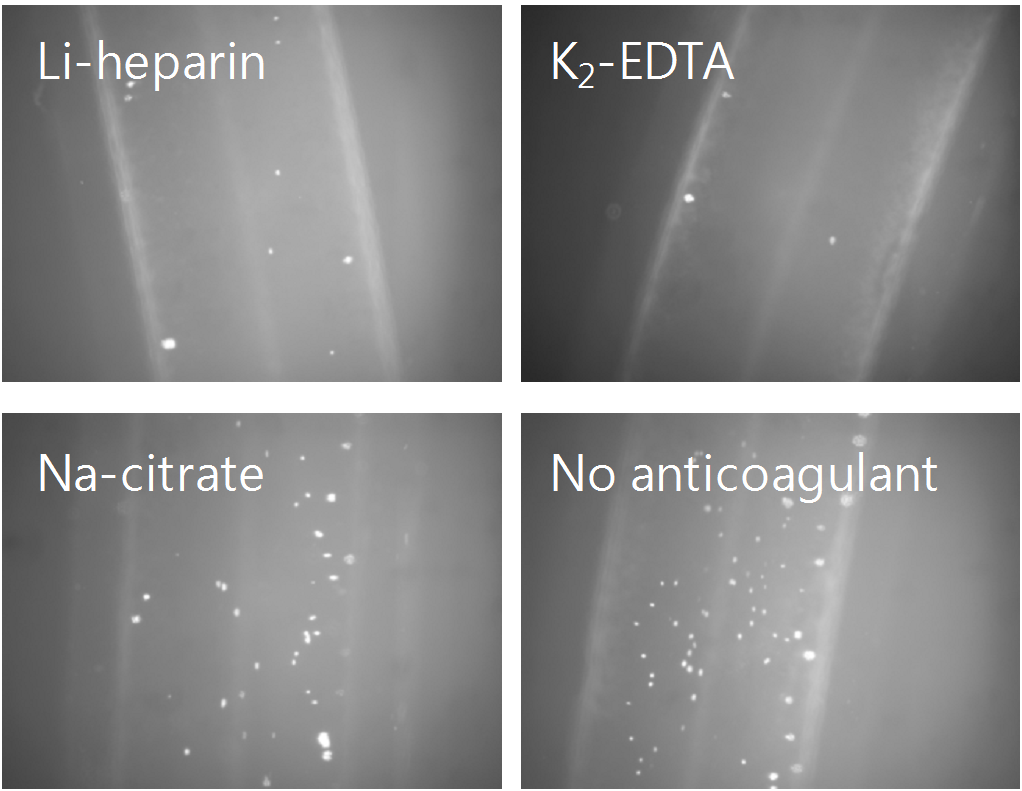

Supplement: S1 Fig — Cell capture was significantly reduced in the blood when Li-heparin and K2-EDTA were used. Successful cell capture was observed in blood with Na-citrate. The cell capturing in the blood without any anti-coagulant was examined by removing early blood clots from blood specimen. The anti-EpCAM immobilized tube could effectively capture the cells in this blood sample. (TIF) [file pone.0133194.s001.tif]

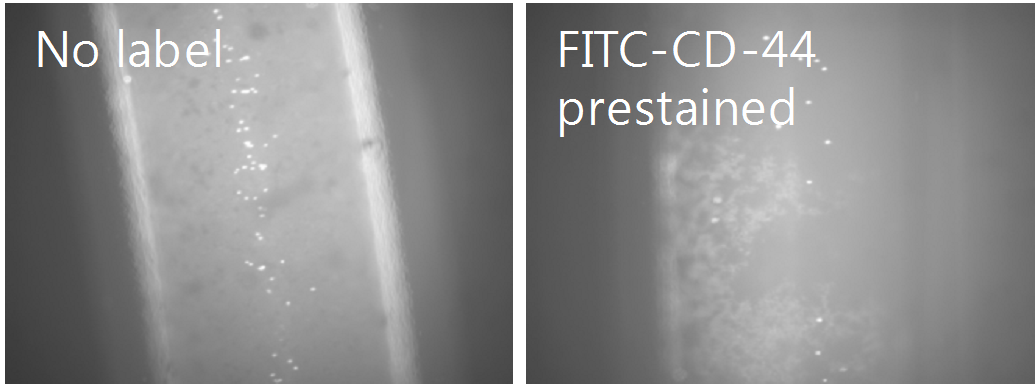

Supplement: S2 Fig — PC-3 cells were labeled by FITC conjugated CD-44 antibodies prior to the cell capturing experiment in the blood. Captured cells were re-stained after capture by filling the tube with Calcein AM contained RPMI media. Compared to PC-3 cell without prior labeling (left), cell capture in CD-44 pre-stained PC-3 was considerably reduced (right). (TIF) [file pone.0133194.s002.tif]
