# Supplementary material for: Chemically Modified Plastic Tube for High Volume Removal and Collection of Circulating Tumor Cells
Source: PLoS One. 2015 Jul 15;10(7):e0133194. doi: 10.1371/journal.pone.0133194 (PMC4503618; doi:10.1371/journal.pone.0133194)
Supplement: S1 Table — (PDF) [file pone.0133194.s003.pdf]

# cell / 100 μL	Initial	Final	Capture efficiency
CD-44 stained PC-3	27,750	22,625	18.5%
PC-3 with no label	29,125	16,62	42.9%
